# Reoptimized UNRES Potential for Protein Model Quality Assessment

**DOI:** 10.3390/genes9120601

**Published:** 2018-12-03

**Authors:** Eshel Faraggi, Pawel Krupa, Magdalena A. Mozolewska, Adam Liwo, Andrzej Kloczkowski

**Affiliations:** 1 Research and Information Systems, LLC, Indianapolis, IN 46240, USA; efaraggi@iupui.edu; 2 Department of Physics, Indiana University Purdue University Indianapolis, Indianapolis, IN 46202, USA; 3 Battelle Center for Mathematical Medicine, Nationwide Children’s Hospital, Columbus, OH 43215, USA; pkrupa@ifpan.edu.pl (P.K.); magdalena.mozolewska@gmail.com (M.A.M.); 4 Institute of Physics, Polish Academy of Sciences, Al. Lotnikow 32/46, PL-02-668 Warsaw, Poland; 5 Institute of Computer Science, Polish Academy of Sciences, ul. Jana Kazimierza 5, 01-248 Warszawa, Poland; 6 Faculty of Chemistry, University of Gdańsk, Wita Stwosza 63, 80-308 Gdańsk, Poland; adam@sun1.chem.univ.gda.pl; 7 Center for In Silico Protein Structure and School of Computational Sciences, Korea Institute for Advanced Study, 85 Hoegiro, Dongdaemun-gu, Seoul 130-722, Korea; 8 Department of Pediatrics, The Ohio State University, Columbus, OH 43215, USA; 9 Kavli Institute for Theoretical Physics China, Chinese Academy of Sciences, Beijing 100190, China; 10 Future Value Creation Research Center, Graduate School of Informatics, Nagoya University, Nagoya 464-8601, Japan

**Keywords:** protein energy, optimization, model ranking, UNRES, Seder, GENN, OUNRES

## Abstract

Ranking protein structure models is an elusive problem in bioinformatics. These models are evaluated on both the degree of similarity to the native structure and the folding pathway. Here, we simulated the use of the coarse-grained UNited RESidue (UNRES) force field as a tool to choose the best protein structure models for a given protein sequence among a pool of candidate models, using server data from the CASP11 experiment. Because the original UNRES was optimized for Molecular Dynamics simulations, we reoptimized UNRES using a deep feed-forward neural network, and we show that introducing additional descriptive features can produce better results. Overall, we found that the reoptimized UNRES performs better in selecting the best structures and tracking protein unwinding from its native state. We also found a relatively poor correlation between UNRES values and the model’s Template Modeling Score (TMS). This is remedied by reoptimization. We discuss some cases where our reoptimization procedure is useful.

## 1. Introduction

The problem of evaluating protein energy and scoring protein conformations has been an important aspect of protein research [1,2,3,4,5,6,7,8,9,10,11,12,13,14,15,16,17,18,19,20,21,22,23,24]. The energy and scoring functions serve to both guide protein simulation studies and to rank putative protein models. There are two main approaches used, categorized as *physical* and *knowledge-based* [25]. In the physical approach, an energy function is built based on a physical model of the atomic interactions and is then optimized based on experimental results. In the knowledge-based approach, the model itself relies on experimental results, typically by matching experimental distributions.

Selecting the best models among putative models is an important application of a protein energy or scoring function [2,3,4,5,6,7,8,9,10,12,13,14,15,16,18,19,20,21,22,23,24]. In such cases, models tailored to a specific sequence are produced, and the task is to rank them according to a specified criterion, usually a measure of the deviation from the native structure corresponding to the given sequence. In this respect, there is a strong overall match between the energy values and the spatial deviation from native scores, such as the Template Modeling Score (TMS) [26]. However, this correspondence is not exact. While energies such as the coarse-grained UNited RESidue (UNRES) force field account for charge distributions, the TMS and similar measures do not. One can imagine transient states arising in cases where the charge distribution has a different transition time than the timescale associated with a structural change. However, protein native and decoy structures are steady states, i.e., they are allowed enough time to relax and escape any unfavorable transient states. Therefore, unstable configurations with lower TMSs but large energies are expected to be excluded. Taking ensemble averages would tend to decrease this effect further.

The current UNRES was optimized to carry out free simulations and not to score decoys [27,28,29]. An attempt at threading was made with a very early version of UNRES [9]; however, even this application involved decoy energy minimization. Given the success of UNRES in free simulations, we considered it worth trying this force field in decoy scoring. It should be noted that free simulations imply that the computed structures are relaxed; if not at all configurations, then at least the end ones. Decoy structures are fixed, and, therefore, clashes from side-chain–side-chain interactions can appear, in general. Consequently, to better design UNRES for decoy scoring, the long-range repulsive components of the potentials need to be better regulated. Thus, a new optimization of UNRES could improve UNRES for decoy scoring. To optimize UNRES for this purpose, we applied a methodology based on neural networks, developed in our earlier work [18,30].

## 2. Methods

### 2.1. UNited RESidue (UNRES)

**Figure 1:**
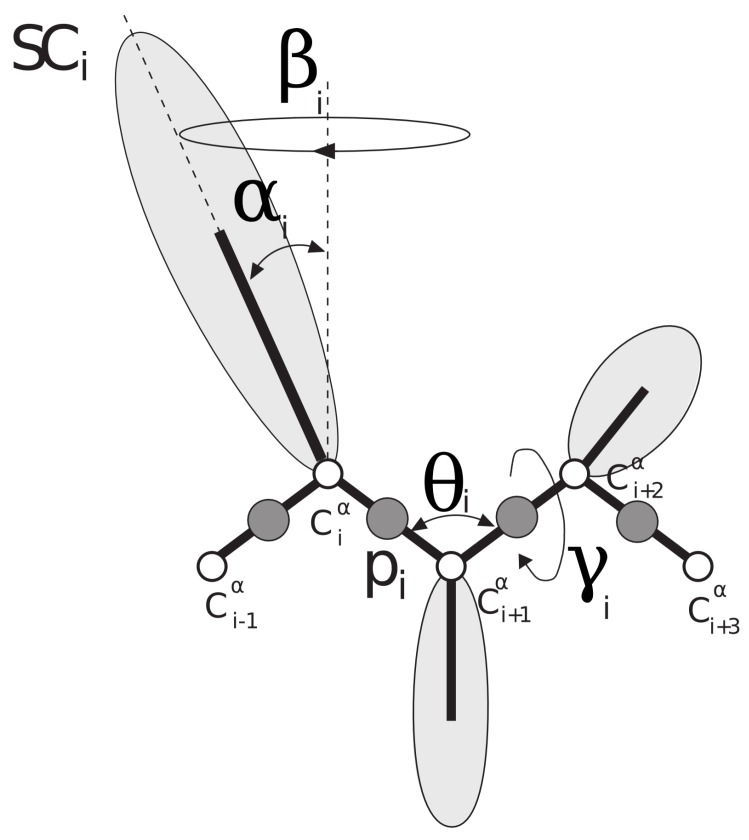
Schematic representation of the UNited RESidue (UNRES) model of the polypeptide chain. There are two interaction sites per residue: united side-chain (SC) and united peptide group (p) are represented by light-gray ellipses and dark-gray circles, respectively. C${}^{\alpha }$ atoms (white circles) and the angles β,α,Θ,andγ define the positions of the backbone and side chains.

UNRES [31,32,33,34] is a coarse-grained model for proteins in which each amino-acid residue is reduced to two interaction sites: the united peptide group (p), located halfway between two consecutive C${}^{\alpha }$ atoms (which are not interaction sites and are only used to define the geometry of the chain), and the united side chain (SC) (Figure 1). Due to the model’s reduced number of interaction sites and the exclusion of averaging out the degrees of freedom (the secondary degrees of freedom), the UNRES force field provides a speed of at least 3 order of magnitude higher compared with the all-atom simulations [35].

The effective energy function in the UNRES model is defined as the restricted free energy (RFE) or the potential of mean force (PMF), and it is given by Equation (1). A detailed description of UNRES is provided elsewhere [36].

(1) $$\begin{array}{c} U=w_{SC}\sum_{i<j}U_{{SC}_{i}{SC}_{j}}+w_{{SC}_{p}}\sum_{i\neq j}U_{{SC}_{i}p_{j}}+w_{pp}^{VDW}\sum_{i<j-1}U_{p_{i}p_{j}}^{VDW}+w_{pp}^{el}f_{2}\left(T\right)\sum_{i<j-1}U_{p_{i}p_{j}}^{el}+\\ w_{tor}f_{2}\left(T\right)\sum_{i<j-1}U_{tor}\left(\gamma_{i}\right)+w_{tord}f_{3}\left(T\right)\sum_{i}U_{tord}\left(\gamma_{i},\gamma_{i+1}\right)+w_{b}\sum_{i}U_{b}\left(\theta_{i}\right)+\\ w_{rot}\sum_{i}U_{rot}\left(\alpha_{{SC}_{i}},\beta_{{SC}_{i}}\right)+w_{bond}\sum_{i}U_{bond}\left(d_{i}\right)+w_{corr}^{\left(3\right)}f_{3}\left(T\right)U_{corr}^{\left(3\right)}+\\ w_{corr}^{\left(4\right)}f_{4}\left(T\right)U_{corr}^{\left(4\right)}+w_{turn}^{\left(3\right)}f_{3}\left(T\right)U_{turn}^{\left(3\right)}+w_{turn}^{\left(4\right)}f_{4}\left(T\right)U_{turn}^{\left(4\right)}+\\ w_{ssbond}\sum_{nss}U_{ssbond}\left(d_{ss}\right)+w_{SC-corr}f_{2}\left(T\right)\sum_{m=1}^{3}\sum_{i}U_{SC-corr}\left(\tau_{i}^{\left(m\right)}\right) \end{array}$$

with

(2) $$f_{n}\left(T\right)=\frac{\ln\left[\exp(1)+\exp(-1)\right]}{\ln\left\{\exp\left[{\left(T/T_{\circ }\right)}^{n-1}\right]+\exp\left[-{\left(T/T_{\circ }\right)}^{n-1}\right]\right\}}$$

where each potential is multiplied by an appropriate weight, $w_{.}$, and these weights are optimized. In Equation (1), $U_{{SC}_{i}{SC}_{j}}$ and $U_{{SC}_{i}p_{j}}$ are side-chain–side-chain and side-chain–peptide-group interaction potentials, respectively. The peptide-group–peptide-group interaction potential is split into the Lennard-Jones term $\left(U_{p_{i}p_{j}}^{VDW}\right)$ and the electrostatic term ($U_{p_{i}p_{j}}^{el}$). The local properties of the polypeptide chain are described by $U_{tor}$, $U_{tord}$, $U_{b}$, $U_{rot}$, and $U_{bond}$ potentials, which are torsional, double torsional, bending, rotameric, and virtual-bond-deformation terms, respectively. $U_{corr}$ and $U_{turn}$ are higher-order correlation terms that are necessary for the correct reproduction of secondary-structure elements [37], $U_{ssbond}$ is a disulfide bond potential, and $U_{SC-corr}$ is a recently implemented potential that couples the local positions of the backbone and side chains, which improves the predictive capacities of the UNRES force field [38,39]. Additionally, because the UNRES energy function originates from the PMF of polypeptide chains in water, in which the fine-grained degrees of freedom have been averaged out, it is temperature-dependent. The factors $f_{i}$ arise from multiplying the terms of the respective order in the cluster-cumulant expansion of the PMF [37]. Because the current implementation of UNRES involves scoring the decoys, which correspond to folded structures and not folding simulations, we set the temperature at T=300 K, assuming that all proteins considered are folded at this temperature. The UNRES model uses an anisotropic potential for the interactions between side chains, which are represented by the Gay–Berne model [40]. This model allows for a more accurate approximation of the side-chain interactions than simpler spherical models.

The energy-term weights in the initial version of the UNRES force field were optimized using only one α-helical protein (PDB code: 1GAB) [27]. We shall term this version of UNRES ‘GB’. In later versions, the force field was re-parameterized using two training mini-proteins: the α-helical tryptophan cage (PDB code: 1L2Y) and tryptophan zipper (PDB code: 1LE1) [28]. The latter force field was recently extended by the addition of the local torsional potentials [39] with very limited manual optimization of the weights of the torsional terms (Table 1). We term this version of UNRES ‘EL’. In the current force field, all the energy terms are physics-based except for side-chain–side-chain interaction terms, which were obtained by an analysis of the PDB [41]. Recently, a new approach to efficient force field optimization was developed [29] based on the maximum likelihood method [42]. However, even with the use of this method, only a very limited number of training proteins can be included in the optimization due to the high computational cost of the iterative procedure based on extensive folding simulations.

For the fold-recognition application reported in this paper, the side-chain–side-chain interaction ($U_{SC_{i}SC_{j}}$), torsional ($U_{tor}$), and side-chain-correlation ($U_{SC-corr}$) terms of Equation (1) are the most important because they account for sequence-specific long- and short-range interactions. Therefore, in addition to the common weights for these terms, we introduced residue-pair-type-specific weights (a total of 400 for each of the three kinds of potentials). Residue-type-specific weights of the excluded-volume contributions ($U_{SC_{i}p_{j}}$) were introduced, because these potentials control the size of the proteins and depend on a single residue type. Likewise, residue-type-specific weights were introduced for the contributions to the virtual-bond-angle ($U_{b}$), side-chain-rotamer ($U_{rot}$), and double-torsional ($U_{tord}$) potentials, the type being that of the central residue. Because, for the decoys taken from the PDB database [41], the regular secondary structure is already present, the electrostatic ($U_{p_{i}p_{j}}^{el}$) and correlation ($U_{corr}^{\left(m\right)}$ and $U_{turn}^{\left(m\right)}$) terms, which determine the regular secondary structure in free simulations, matter only as much as they contribute to the energy of the “bulk” of the secondary structure of different types. Therefore, only the weights corresponding to total $U_{p_{i}p_{j}}^{el}$, $U_{p_{i}p_{j}}^{VDW}$, $U_{corr}^{3}$, $U_{corr}^{4}$, $U_{turn}^{3}$, and $U_{turn}^{4}$ were optimized (one weight per each kind of term).

Multiple types of calculations can be performed with UNRES, from single-point energy calculations and energy minimization in internal and external coordinates to Monte Carlo and Molecular Dynamics calculations of various variants and modifications. Including serial (sequential) and parallel runs with scaling up to 70% on 16K cores [43]. Example of UNRES usage include: Conformational Space Annealing (CSA) [44], Hybrid Monte Carlo (HMC) [45], Replica Exchange Molecular Dynamics (REMD) [46], and Multiplexed Replica Exchange Molecular Dynamics (MREMD) [47]. UNRES has been successfully used for studies of protein folding pathways, thermodynamics, and kinetics [48,49,50]; in studies of multimeric systems [51,52] with the use of periodic boundary conditions [53]; and in systems with nonstandard amino acids [54] and links [55]. More detail on UNRES can be found elsewhere [36].

### 2.2. Reoptimization

As stated in the introduction, the current version of UNRES was optimized to run free simulations in which the potential clashes are removed; however, in general, this is not the case for scoring fixed decoys. Therefore, first, we modified the potential to limit its repulsive components. Specifically, we imposed a cutoff on the repulsive parts of the potential to limit the maximum repulsion to 3 kcal/mol for a given interaction type between each pair of interaction centers. Only with such an approach can the UNRES force field be used without the prior energy minimization of a system because, otherwise, even slight overlapping of the interaction centers can outweigh all other energy components. Another possibility would be to use soft potentials, such as the 8–6 Lennard-Jones potential, but, even then, short energy minimization is needed [56]. We then reoptimized the force field to accommodate this change and to customize it to the task of decoy scoring.

We employed the neural network technique for optimization. In the implementation of the UNRES model developed in this work, although energy is a linear function of the parameters, the error function to minimize is expressed as:

(3) $$F=\sum_{p=1}^{totp}\sum_{i=0}^{N_{p}-1}{\left[TMS_{p}^{pred}\left({\left\{U\right\}}_{pi}\right)-TMS_{pi}^{decoy}\right]}^{2}$$

where index *p* runs over the training proteins, index *i* runs over the decoys corresponding to a given training protein (including the native structure, which has an index of 0), totp is the total number of training proteins, $N_{p}$ is the total number of decoys for protein *p* (including the native structure of this protein), $TMS^{pred}$ and $TMS^{decoy}$ denote the predicted TM-scores and those calculated from the respective decoy and native structures, and the input features are ${\left\{U\right\}}_{pi}$, the set of UNRES energy components (Equation 1) calculated for decoy *i* of protein *p*. As described later, other input features will be used alongside ${\left\{U\right\}}_{pi}$. In this work, we used the back-propagation neural network method to approximate the values of $TMS_{p}^{pred}$ that will minimize Equation (3). The neural network we used is a nonlinear function from the input features (${\left\{U\right\}}_{pi}$) to the output feature ($TMS^{pred}$). We started with a random set of neural network weights and passed the input features through the neural network weights to calculate $TMS^{pred}$. This part of the process is known as feed-forward. We then calculated the error associated with said prediction and, by the steepest descent method, modified the neural network weights to reduce this error. This process is known as back-propagation. It was carried out repeatedly until an overfit-protection test was violated. In this case, for the overfit protection, we left out part of the data from training and chose weights that gave the best results for this left-out set.

From UNRES, we first extracted information characterizing the state of the protein as an initial step toward UNRES reoptimization. Besides giving the overall UNRES energy value and the values for each of the components in Equation (1), we also split these components into their residue-type-specific contributions. For example, for paired interactions, we calculated separate values for the contributions from interactions between one type of residue and another. In total, we have the following input features from UNRES: one overall energy, nine single-value components, four 20-valued components that are residue-type-dependent, and three 400-value components that are dependent on the type of a residue pair. See Table 2 for a list and description of these labels. Additionally, the weights of each kind of UNRES energy component and that of the total UNRES energy were also optimized. In total, we have 1+15+4×20+3×400=1296 values characterizing the UNRES energy function in this study. It should be noted that the parameters of the pairwise side-chain–side-chain interaction energies are not symmetric. The reason for this is the directionality of the protein chain (from the N to the C terminus). For example, a parameter for an ‘AC’ pair of side chains means that alanine precedes cysteine in sequence. Such non-symmetry of interactions is quite commonly used in fold-recognition studies [1,2,3,4,5,6] and partially accounts for the “through sequence” long-range interactions.

The same approach we used in the Seder1 scoring function [18] was used here for scoring a model of a given sequence based on its similarity to the native PDB [41] structure of the sequence, as measured by the TMS, which provides a normalized value for training our networks. In addition, we established a formula for transforming the TM-score, $TMS^{\prime }=1-2\times TMS$, to achieve a distribution of values more fitting to our bipolar selection of neural networks and to align the directionality between our score and the energy values. With our transformation, a native structure scores a ‘−1’.

**Figure 2:**
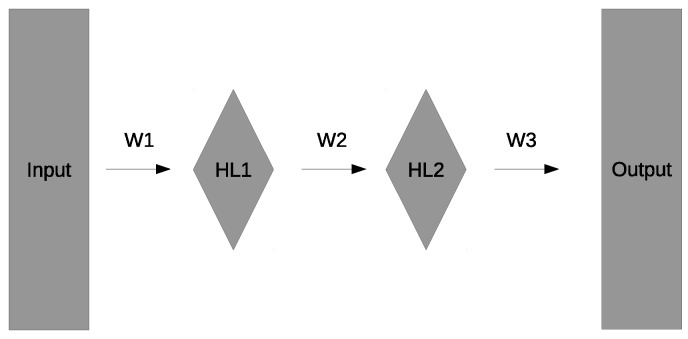
General architecture of the neural network. HL1 and HL2 refer to the first and second hidden layers, respectively, and W1, W2, and W3 refer to the weights connecting the different layers.

We used a two-layer feed-forward neural network with momentum, recently described in detail [30]. A diagram of the neural network architecture is given in Figure 2. HL1 and HL2 refer to the first and second hidden layers, respectively, and W1, W2, and W3 refer to the weights connecting the different layers. We used an all-connected network in which weights connect all the nodes of one layer to all the nodes of the next layer. The number of weights for a given network will depend on the number of inputs, as this will determine the number of weights in W1. For an all-connected network, the number of weights connecting layer L1 with $h_{1}$ nodes (plus a bias node) to layer L2 with $h_{2}$ nodes is $\left(h_{1}+1\right)\times h_{2}$. At each node, the weighted sum of the previous layer is passed through an activation function to give the value of that node. We used a bipolar hyperbolic tangent activation function. Momentum refers to the contribution of the gradient calculated at the previous time step to the correction of the weights.

We used the steepest-descent back-propagation algorithm to optimize the neural network weights. We started the analyses with a randomly selected training set of 22,805 protein chain models from the full training set of 296,381. The use of a small training set enabled us to optimize the architecture. After that initial optimization, deviations from the optimized values were tested for the full set. The final optimized values are given in Table 3. From the full training set, we selected 30% of the proteins at random for an overfit-protection set. A total of six such random training/overfit sets were used to train different realizations of the neural network. For each of the approaches to optimizing UNRES in Table 4, the initial weights and the order of the training proteins were randomized for each of the six neural network realizations used for the corresponding approach. We chose to use six realizations based on experience from previous work [18,30,57,58]. To obtain the final prediction, we averaged the results from the six realizations. This also gives an estimate for the stability of the prediction through the standard deviation. We obtained the full training and overfit-protection sets from three sources (number of models given in parenthesis): server models submitted to CASP4 through CASP10 (123,634) [59], native models from the PDB (54,084) [41], and native models from the UCSF database of protein models (118,663) [60]. The three sources are treated in more detail in our previous publication [18]. Combining these three sources resulted in 296,381 proteins. From these, we randomly set aside 30% for the overfit-protection set and used the rest for training. We used the published results from CASP11 [59] as the testing set for the results here. This set contains 83 proteins that were selected by the CASP organizers to represent a variety of protein structures. Our approach here simulates participation in CASP11.

Both the EL and GB versions of UNRES were optimized, resulting in the OUNRES (Optimized UNRES) versions. We also tried to integrate additional external information into UNRES, such as the number of residues, the number of atoms, and the scores from DFire2 [61] and Seder1 [18]. All input features were z-scored. We used cutoff values, defined by the values of the top and bottom 1% of the data, to limit the effect of outliers in the UNRES values.

Our testing method consisted of simulating participation in the CASP11 competition [59]. All of our training instances were restricted to models available before the release of CASP11 targets. We collected a total of 83 surviving CASP11 targets and the top 150 server predictions for them. This resulted in a total testing dataset of 12,240 structures. No information from these structures, such as overfit protection, parameter optimization, or any others, was used in the training of the neural network for the testing reported here. However, the version of OUNRES released with this work (and available from http://mamiris.com/services.html) used CASP11 information to select the top-performing networks among possible candidates.

## 3. Results

We analyzed the results of the optimization of the partial UNRES energies for both the GB and EL methods, with and without added input features. Protein structure scoring functions have several uses of interest. Among these functions is the ability to select the closest model to a native structure and, in the scope of protein folding, to select models along the folding pathway that approach a native state. Testing the first application of selecting the model closest to the native structure is relatively easy; testing the second use is considerably more challenging. We used the Pearson correlation with the real TMS and several other self-developed methods to estimate the effectiveness of selecting paths along the folding pathway; however, these results are more difficult to interpret since TMS does not account for charge distributions, as mentioned earlier.

We began by comparing the mean TMS of the top five selected models according to the various methods. We first ranked the models according to the prediction of a given method and then calculated the mean TMSs of the top five models for each of the 83 CASP11 targets. The mean and standard deviation (STD) of these 83 values were calculated for each method. The results of these calculations are given in Table 4.

The results of the correlation were also calculated. Pearson correlations were calculated between the TMS to native structure of a model and its prediction according to the different methods. The correlation was also calculated between the TMS to native and the UNRES energy. This calculation was done per target. Then, the mean and STD of the correlations calculated for each of the 83 targets were obtained. These results are presented in Table 5. The Pearson coefficients are low, but it should be noted that the coefficients obtained for the OUNRES variants are higher than those for Seder1 and DFire2 alone; this means that OUNRES can rank the bulk of the decoys better than those two methods.

**Figure 3:**
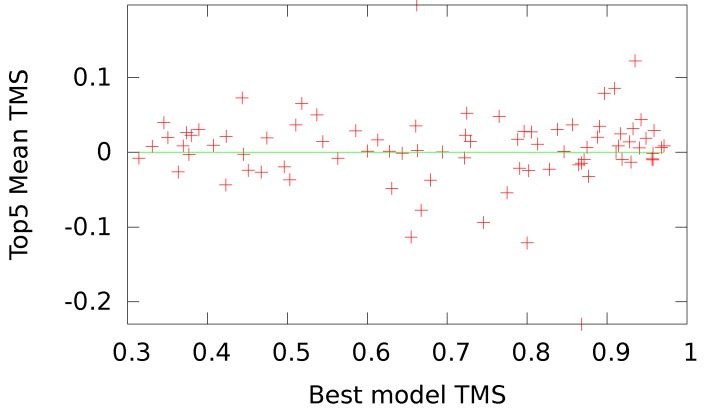
Difference in the top five mean TMSs between OUNRES and UNRES as a function of the TMS to native of the best available decoy for that target submitted during the CASP11 experiment. Smaller values on the x-axis indicate harder targets, while the y-axis measures the success of UNRES optimization, with higher values indicating greater success.

**Figure 4:**
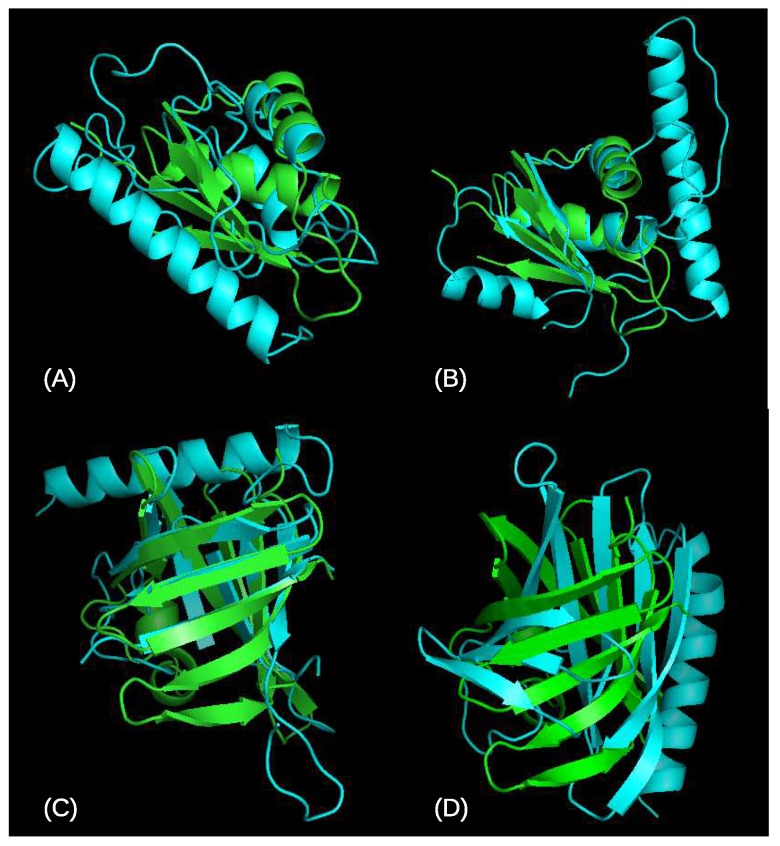
Structures for the top scored models for the two worst cases in Figure 3 using UNRES and OUNRES. Native structures are given in green, decoy model structures in cyan. (**A**) UNRES best for target T0765, model Alpha-Gelly-Server-TS5; (**B**) OUNRES best for target T0765, model RBO-Aleph-TS3; (**C**) UNRES best for target T0782, model SAM-T08-server-TS2; (**D**) OUNRES best for target T0782, model RBO-Aleph-TS3.

**Figure 5:**
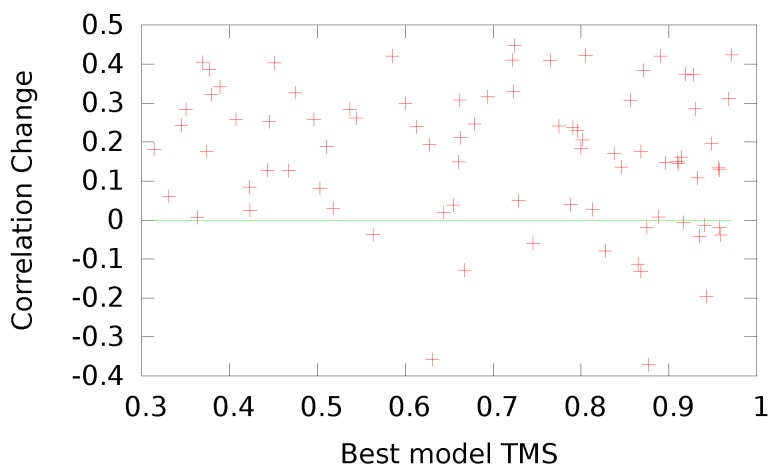
Difference in correlation between OUNRES and UNRES as a function of the TMS to native of the best available decoy for that CASP11 target. Smaller values on the x-axis indicate harder targets, while the y-axis measures the success of UNRES optimization, with higher values indicating greater success.

To better understand the result of our optimization, in Figure 3 we give the differences in the top five mean TMSs between OUNRES and UNRES as a function of the TMS to native of the best available decoy for that target (topTMS). In most cases (51/83), with both easy and hard targets, we find that OUNRES is an improvement over UNRES. In some cases, the optimization seems to reduce the quality of the top selected models, represented by a highly negative y-axis. We looked at the two worst cases: T0782 with a topTMS 0.85 and T0765 with a topTMS of 0.8. In both cases, OUNRES appears to perform significantly worse than UNRES, judging by the top five mean TMSs. If we observe the resulting protein structures in Figure 4, we see that in the case of T0782, UNRES seems to better model the beta barrel. However, in the case of T0765, it seems that OUNRES produces a better structure, while the increase in TMS for UNRES is mostly due to the structure being more compact. In Figure 5, we plot the change in correlation upon optimization as a function of the topTMS. We see that the correlation improves for most (68/83) targets. Additionally, it seems that only targets with a high topTMS (easy targets) are made worse by using OUNRES over UNRES. For hard targets, it seems that the correlation is always improving.

We also tested the directional accuracy of the different methods in two ways. First, we calculated the mean TMS for the top 1–5 (top1-5) and top 10–15 (top10-15) real model TMS to native ranking. We then calculated the average score/energy for the top1-5 and top10-15 sets according to the different methods and calculated the change. If the change was appropriate for a given score/energy, i.e., it points to the top1-5 being more favorable, we assigned a ’+1’ for this CASP11 target. If the score was inappropriate, we assigned a ’−1’ to this target. We then calculated the average assignment of the 83 targets and the STD. We term this parameter the *Directional Accuracy* (DA). Results for the DA are presented in Table 6.

This test can also be done in the reverse order. The models can be ranked according to a score/energy, then the real TMS difference between the top1-5 and the top10-15 can be calculated. We calculated this for the 83 CASP11 targets we used and averaged the results to arrive at a single value per method employed. We also calculated the STD for this test. We term this parameter the *Second Directional Accuracy* (DA2). Results for DA2 are given in Table 7.

We also tested a path to native accuracy. We can imagine that the server models we collected from the CASP11 experiments are a folding pathway in the configuration space to the native structure. To obtain this pathway, we started with the native model for a given target sequence. We then found the nearest structure, as measured by the real TMS, and repeated the process. The used structures were excluded until all models for the target were exhausted. Following the consecutively closest structures, a folding pathway was obtained, for which energies and scores were calculated. In a similar fashion to that above, we assigned a ‘+1’ if the change in energy or score was consistent with the direction of the path, i.e., decreasing or not changing as the native state is approached, and a ‘−1’ otherwise. We then averaged these values along the path for a given CASP11 target and then averaged the resultant means to arrive at a single value per method. We call this the *Path to Native Accuracy* or PNA. These results are given in Table 8.

## 4. Discussion

We see an overall consistent improvement with the optimization and the addition of input features across all tests undertaken. We did not find any significant advantage of the EL or GB approaches to UNRES over the other. For the mean TMS of the top1-5 models, we see a consistent improvement, with the optimization adding about a percent of relative accuracy and inclusion of the sequence length and number of atoms adding another relative percent to the accuracy. Improvements over UNRES of OUNRES+length (EL or GB) have a statistical confidence of more than 99% according to a two-sided Student’s t-test. The addition of information from DFire2 does not seem to improve the accuracy for this case.

We also calculated the mean TMS for the top five ranked structures using only DFire2 or Seder1 to perform the ranking. In both cases, we get slightly better results than the neural networks trained on parameters extracted from UNRES, with and without Seder1 or DFire2 or both as inputs. This seems to be due to the optimization of the scoring for models farther from the native state than the top five. This can be seen in terms of correlations, where using either Seder1 or DFire2 yields worse correlations, with Seder1 outperforming DFire2 both for correlations and for top five mean TMS. These observations seem to indicate that although we seem to have improved UNRES by optimization, there is information yet to be picked up by the neural network for close to native structures.

A significant effect is observed for the Pearson correlation, where a 2-fold increase is observed upon the optimization of UNRES. Slight fluctuation around this improvement is observed with the introduction of additional input features; however, there is no significant improvement above a correlation of 0.32–0.33. As indicated earlier, the strong improvement upon optimization could be in part due to UNRES’s consideration of charge distributions and the fact that the correlation is calculated over the entire sample of models for a given target.

For DA1 and DA2, we again see a strong response upon optimization and some additional response to the inclusion of additional input features. In both cases, we expect that the better the score/energy, the more consistently appropriate will be the change in values between the top1-5 to top10-15 models. More than a 2-fold increase in accuracy is observed upon optimization, and an additional significant improvement in accuracy is observed if additional input features are introduced. Improvement due to optimization in both cases has a confidence greater than 99% according to a two-sided Student’s *t*-test. One should note that due to the choice of variables for test DA1 (1,−1) and its discrete nature, the STD in this case is exaggerated. Note that for DA2, for the optimized methods, the signal is almost entirely positive; i.e., the mean minus the STD is greater than zero. This indicates that the optimized methods were directionally successful for almost all protein targets. For the most successful method, OUNRES+length, 72 out of 83 targets had the correct directionality.

**Figure 6:**
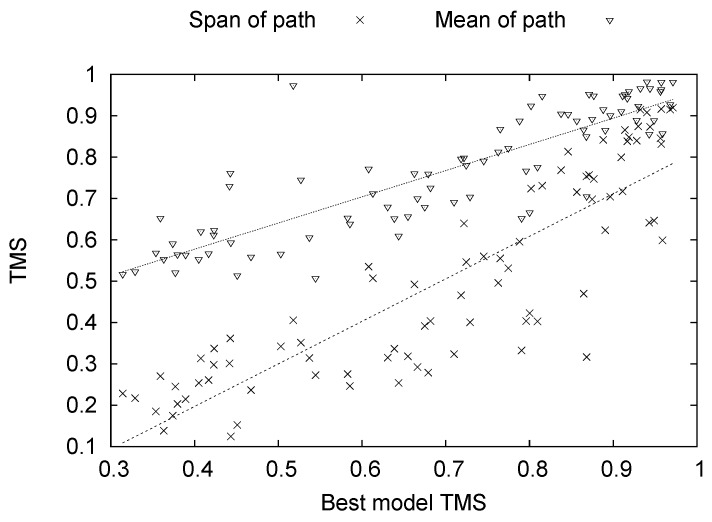
Mean (triangles) and span (x) of the path as a function of the best server model TMS submitted to CASP11. The Pearson correlation coefficients between the best model TMS and the mean/span of the path are 0.868/0.869, respectively. The correlation between the mean and the span of the path is 0.922. The best fit lines are presented to aid the eye. The line for the span of the path is given by l(x)=1.03x−0.21. The line for the mean of the path is given by l(x)=0.63x+0.32.

For the PNA, we do not see a strong signal. The fluctuations in the PNA are quite large, indicating that, for many targets, the directional assignments were rendered more erroneous. However, there is enough signal to observe a significant improvement from the optimization and additional input features. In this case, there is no clear consistency in the improvements though, and that could be due to the nature of the path to the native structure. It is interesting to note that the path to native is intimately related to the hardness of the target. In Figure 6, we give the mean and span of the path as a function of the best model TMS submitted to CASP11. The 83 points in the plot correspond to the 83 CASP11 targets we used. The mean of the path is defined as the average over the TMS between consecutive models along the path. The span of the path is defined as the TMS between the native structure and the final model structure in the path until all others have been excluded. The Pearson correlation coefficients between the best model TMS and the mean/span of the path are 0.868/0.869, respectively. The correlation between the mean and the span of the path is 0.922. We also calculated the best fit lines between the best model TMS and the mean/span of the path. The line for the span of the path is given by l(x)=1.03x−0.21. The line for the mean of the path is given by l(x)=0.63x+0.32.

## 5. Conclusions

We reoptimized the UNRES energy function for protein decoy model quality assessment and achieved consistently better results in a number of tests. We find the bulk of the improvement for this round of optimization is from the improved scoring for the bulk of the models. This is seen by the large increase in the correlation of OUNRES relative to UNRES. This bias toward improving the bulk of the data results from the choice of neural network architecture and approach. It should be noted that this is the first attempt at using UNRES for scoring fixed decoy sets. A very early version of UNRES was used for threading [9], but in that work, the decoys were subjected to restrained energy minimization with UNRES, and minimized energies were used for scoring.

We introduced several quantities to help compare the energy/score functions of proteins. The Top5TMS measures the average TMS to native of the top five picked by the method. DA1 and DA2 provide a measure of the directional successes of the energy/score function in terms of the path to native. Finally, the PNA is a measure of the directional success of an energy/score function along the folding pathway of a protein.

We find that additional information in the form of additional input features tends to improve the accuracy of UNRES and OUNRES in picking the closest to native models and in assigning the direction toward a closer to native structure. In this respect, it seems that simply adding the z-scaled number of residues and atoms improves the performance of OUNRES most significantly. However, we find that DFire2 does not seem to improve the performance of UNRES, possibly due to an existing PMF in UNRES. On the other hand, the DA1, DA2, and PNA measures suggest that OUNRES has a substantial power of energy-ranking the bulk of the decoys and can, therefore, be used for selecting the decoys for further processing, rather than picking prediction candidates. Thus, OUNRES seems to be of advantage when decoys need to be pre-selected for further processing, rather than for the selection of the final models.

## Figures and Tables

**Table 1:** Optimized weights for the EL variant of UNRES.

Weight Type	Value
$w_{SC}$	1.00000
$w_{{SC}_{p}}$	1.23315
$w_{pp}^{VDW}$	0.23173
$w_{pp}^{el}$	0.84476
$w_{tor}$	1.34316
$w_{tord}$	1.26571
$w_{b}$	0.62954
$w_{rot}$	0.10554
$w_{bond}$	1.00000
$w_{corr}^{\left(3\right)}$	0.37357
$w_{corr}^{\left(4\right)}$	0.19212
$w_{turn}^{\left(3\right)}$	1.40323
$w_{turn}^{\left(4\right)}$	0.64673
$w_{ssbond}$	1.00000
$w_{SC-corr}$	0.25000

The standard energy-term weights used in the UNRES energy function. These optimized weights correspond to the EL variant of UNRES. Abbreviations: Side Chain (SC), Van Der Waals (VDW), electrostatic (el), torsion (tor), toroidal (tord), bending (b), rotation (rot), bonding (bond), correlation (corr), turns (turn), and secondary structure bond (ssbond).

**Table 2:** Labels for UNRES characterization.

Label	Size	Description
$U_{SC_{i}SC_{J}}$	400	Side-chain hydrophobic (hydrophilic) interaction
$U_{SC_{i}p_{j}}$	20	Excluded-volume side-chain–peptide-group interactions
$U_{p_{i}p_{j}}^{vdW}$	1	Lennard-Jones interaction energy between peptide-group centers
$U_{pipj}^{el}$	1	Electrostatic energy between peptide-group dipoles
$U_{tor}$	400	Virtual-bond dihedral angle torsional terms
$U_{tord}$	20	Virtual-bond dihedral angle double-torsional terms
$U_{b}$	20	Virtual-bond angle bending terms
$U_{rot}$	20	Side-chain rotamer term
$U_{bond}$	1	Virtual-bond-deformation term
$U_{corr}^{\left(3\right)}$	1	Third-order correlation term
$U_{corr}^{\left(4\right)}$	1	Multibody coupling backbone-local and backbone electrostatic interactions
$U_{turn}^{\left(3\right)}$	1	Correlation contributions involving three consecutive peptide groups
$U_{turn}^{\left(4\right)}$	1	Correlation contributions involving four consecutive peptide groups
$U_{ssbond}$	1	Residue-dependent side-chain rotamer terms
$U_{SC-crr}$	400	Physics-based side-chain backbone correlation potentials

Labels for the energy decomposition used as input for representing UNRES to the neural network and their description. Size refers to the number of components a given label has. Some, such as the overall energy, are single-valued (1), four depend on the residue type and are 20-valued (20), and three depend on the type of a residue pair and are 400-valued (400).

**Table 3:** Optimized parameters for neural networks.

Parameter	Value
HL1	6
HL2	7
*a*	0.2
μ	0.003973
*P*	0.4
$N_{max}$	10,000
$N_{stop}$	400

HL1 and HL2 are the number of neurons in the first and second hidden layers, respectively. An additional bias neuron is used in each layer. *a*, μ, and *P* are the activation parameter, the learning rate, and the momentum, respectively. $N_{max}$ and $N_{stop}$ are the maximum number of epochs and the number of epochs necessitating a stop after no improvement on the overfit-protection set, respectively.

**Table 4:** Top five mean Template Modeling Scores (TMSs).

EL			GB		
Method	Top5TMS	STD	Method	Top5TMS	STD
UNRES	0.596	0.074	UNRES	0.596	0.074
OUNRES	0.599	0.076	OUNRES	0.601	0.068
OUNRES+Seder1	0.600	0.072	OUNRES+Seder1	0.600	0.079
OUNRES+length	0.605	0.076	OUNRES+length	0.605	0.077
OUNRES+both	0.606	0.073	OUNRES+both	0.606	0.079
OU+both+DFire2	0.605	0.081	OU+both+DFire2	0.603	0.077
DFire2 alone	0.607	0.074	Seder1 alone	0.611	0.085

EL and GB UNRES variants tested relative to Optimized UNRES (OUNRES), OUNRES with Seder1 as neural network input (OUNRES+Seder1), OUNRES with residue length and number of atoms (OUNRES+length), OUNRES with both Seder1 and length (OUNRES+both), and OUNRES with Seder1, length, and DFire2.0 (OU+both+DFire2). Top5TMS is the mean of the TMSs of the top five models selected by each method. STD is the standard deviation and was calculated over the 83 CASP11 targets.

**Table 5:** Pearson Correlation.

EL			GB		
Method	Correlation	STD	Method	Correlation	STD
UNRES	0.151	0.189	UNRES	0.150	0.177
OUNRES	0.322	0.173	OUNRES	0.315	0.176
OUNRES+Seder1	0.320	0.182	OUNRES+Seder1	0.326	0.186
OUNRES+length	0.316	0.201	OUNRES+length	0.332	0.194
OUNRES+both	0.327	0.175	OUNRES+both	0.326	0.195
both+DFire2	0.320	0.186	both+DFire2	0.334	0.199
DFire2 alone	0.247	0.215	Seder1 alone	0.280	0.185

Refer to Table 4 for the legend. STD is the standard deviation and was calculated over the 83 CASP11 targets.

**Table 6:** Directional Accuracy (DA).

EL			GB		
Method	DA	STD	Method	DA	STD
UNRES	0.084	0.996	UNRES	0.084	0.996
OUNRES	0.181	0.984	OUNRES	0.181	0.984
OUNRES+Seder1	0.253	0.967	OUNRES+Seder1	0.229	0.973
OUNRES+length	0.253	0.967	OUNRES+length	0.157	0.988
OUNRES+both	0.181	0.984	OUNRES+both	0.205	0.979
both+DFire2	0.181	0.984	both+DFire2	0.205	0.979

Refer to Table 4 for the legend. STD is the standard deviation and was calculated over the 83 CASP11 targets. Note that it is artificially high because of the discrete nature of this test.

**Table 7:** Second Directional Accuracy (DA2).

EL			GB		
Method	DA2	STD	Method	DA2	STD
UNRES	−0.009	0.065	UNRES	−0.008	0.067
OUNRES	0.058	0.073	OUNRES	0.058	0.076
OUNRES+Seder1	0.064	0.070	OUNRES+Seder1	0.056	0.068
OUNRES+length	0.064	0.074	OUNRES+length	0.070	0.068
OUNRES+both	0.061	0.078	OUNRES+both	0.066	0.066
both+DFire2	0.061	0.083	both+DFire2	0.065	0.074

Refer to Table 4 for the legend. STD is the standard deviation and was calculated over the 83 CASP11 targets. Note that, in this case, for the optimized methods, the signal is almost entirely positive, which indicates that the methods were directionally successful for almost all protein targets (72/83 for OUNRES+length).

**Table 8:** Path to Native Accuracy (PNA).

EL			GB		
Method	PNA	STD	Method	PNA	STD
UNRES	0.017	0.051	UNRES	0.019	0.057
OUNRES	0.028	0.051	OUNRES	0.020	0.057
OUNRES+Seder1	0.018	0.054	OUNRES+Seder1	0.010	0.052
OUNRES+length	0.034	0.053	OUNRES+length	0.024	0.046
OUNRES+both	0.023	0.049	OUNRES+both	0.013	0.047
both+DFire2	0.026	0.055	both+DFire2	0.021	0.059

Refer to Table 4 for athe legend. STD is the standard deviation and was calculated over the 83 CASP11 targets.
